# Unusual Sternoclavicular Joint Infection With Pseudomonas in a Young Adult With Hidradenitis Suppurativa: A Case Report

**DOI:** 10.7759/cureus.101004

**Published:** 2026-01-07

**Authors:** Perla E Abboud, Mariebelle C El Khoury, Demy G Batanian, Jean Claude Kheirallah

**Affiliations:** 1 Internal Medicine, Lebanese University, Faculty of Medical Sciences, Beirut, LBN; 2 Internal Medicine and Infectious Diseases, Lebanese Hospital Geitaoui, Beirut, LBN

**Keywords:** cellulitis, hidradenitis suppurativa, osteomyelitis, pseudomonas aeruginosa, sternoclavicular joint infection

## Abstract

Sternoclavicular joint infection is a relatively rare and serious condition, and its association with *Pseudomonas aeruginosa* is uncommon. Moreover, hidradenitis suppurativa is a chronic inflammatory skin disease that may predispose patients to deep-seated infections, as illustrated by this unusual report. We present a case of a 31-year-old male patient with a *Pseudomonas aeruginosa* sternoclavicular joint infection. The subject presented for a three-month history of swollen, erythematous, and tender right sternoclavicular joint, refractory to non-steroidal anti-inflammatory drugs (NSAIDs) and steroid injections. As past medical history, the patient has hidradenitis suppurativa that was quiescent at the time of presentation. MRI of the right shoulder showed findings suggestive of osteomyelitis and cellulitis. Soft tissue culture showed a *Pseudomonas aeruginosa* infection, and blood cultures yielded no growth. He showed clinical improvement and normalization of inflammatory markers with intravenous ceftazidime for two weeks, and he was discharged home on oral ciprofloxacin for four weeks. In this context, we underscore the need to consider uncommon pathogens in atypical infection sites.

## Introduction

Although rare, the sternoclavicular joint infection (SCJ) carries substantial clinical relevance, given its close anatomical proximity to major neurovascular structures such as the phrenic nerve and subclavian vessels. Its potential complications range from a simple abscess to mediastinitis and even sepsis [1]. Septic arthritis of the sternoclavicular joint is often difficult to diagnose at the first clinical encounter, and it may evolve into osteomyelitis in case of delayed diagnoses and suboptimal initial therapy. It typically presents with acute unilateral onset of pain, fever, warmth, swelling, and shoulder immobility, and predominantly affects the right side [1,2]. The most common underlying risk factors involve diabetes mellitus, intravenous drug use, intra-articular injections, rheumatoid arthritis, trauma, immunosuppression, end-stage renal disease, and the presence of a central venous catheter [3]. The most common organism associated with sternoclavicular joint infection is *Staphylococcus aureus* [4]. Although uncommon, Pseudomonas infection of the sternoclavicular joint is primarily associated with intravenous drug use [5].

Hidradenitis suppurativa (HS) is a chronic multifactorial inflammatory skin disease that primarily affects intertriginous areas, including the axillary, inguinal, submammary, and anogenital body regions [6]. It is characterized by the occlusion of pilosebaceous units manifesting as tender nodules, abscesses, and draining sinuses, and may result in disfiguring scars [7]. Furthermore, hidradenitis suppurativa exhibits elevated levels of proinflammatory cytokines, including interleukin-17 and tumor necrosis factor, which explain its association with systemic inflammatory diseases and inflammatory arthritis, notably spondyloarthritis [6]. Additionally, the presence of diabetes mellitus, smoking, obesity, and poorly controlled HS increases susceptibility to infectious complications [6,7]. The most commonly isolated pathogens from HS lesions are *Staphylococcus*, *Streptococcus*, *Corynebacterium*, *Escherichia-Shigella*, and *Porphyromonas* [8].

## Case presentation

**Figure 1:**
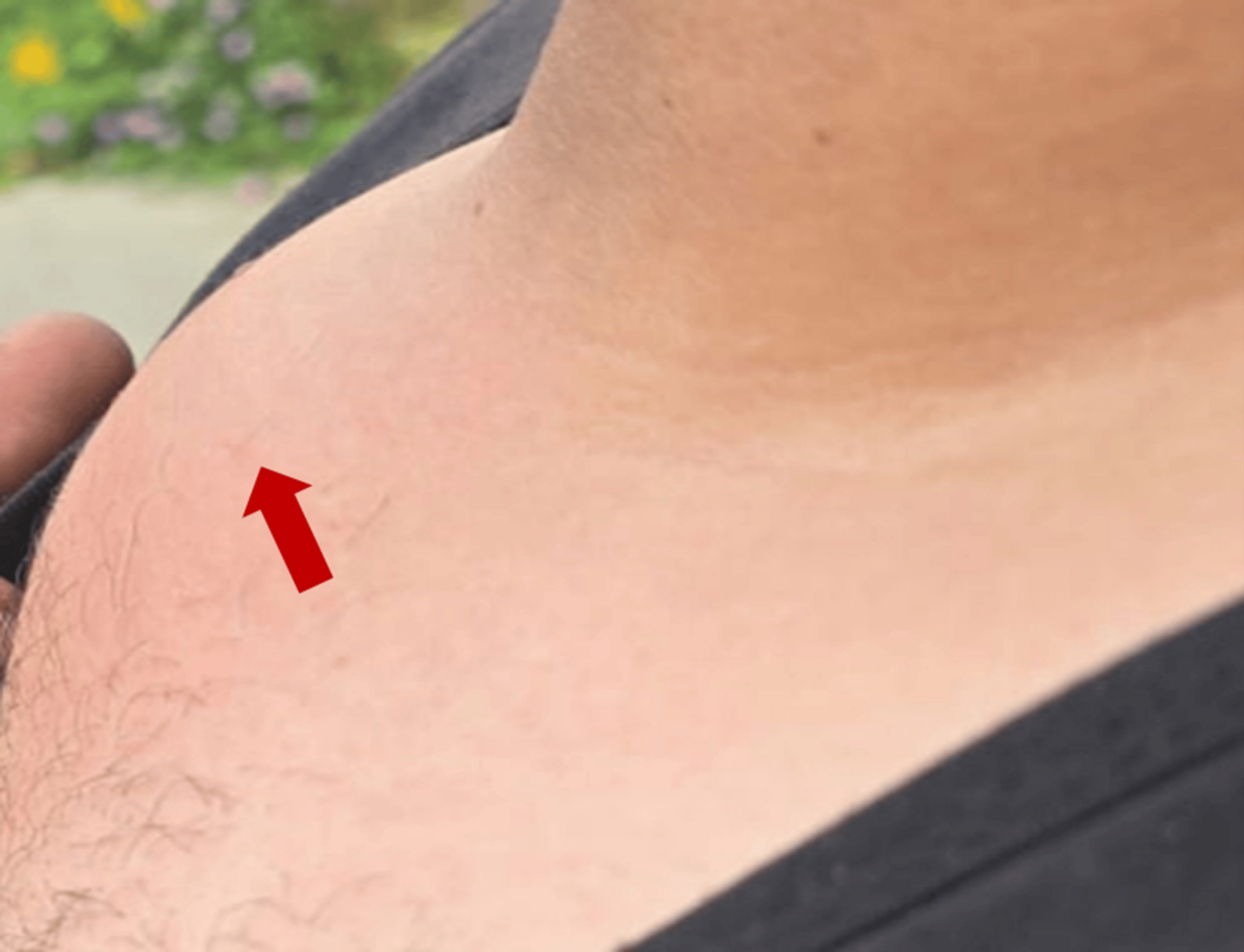
Swelling at the right sternoclavicular junction (arrow)

A 31-year-old male patient presented to our hospital for a 3-month history of a swollen, erythematous, warm, and tender right sternoclavicular joint, right shoulder immobility, and chills (Figure 1). Despite multiple therapeutic regimens, including steroid injection, the patient reported no major improvement. At first, he received oral and intramuscular non-steroidal anti-inflammatory drugs (NSAIDs) over several weeks. He subsequently completed a 10-day course of oral cefuroxime without any improvement. His past medical history includes axillary Hidradenitis suppurativa managed with a topical therapy, and the patient had residual scarring at the time of presentation, as active lesions had subsided. The patient underwent gastroscopy and colonoscopy one year prior, and findings were remarkable for peptic ulcer disease. He was a smoker, consumed alcohol occasionally, and denied any illicit drug use.

On presentation, the patient was afebrile, and vital signs were within normal limits. The right sternoclavicular joint was swollen, tender, erythematous, and warm on physical examination in association with a decreased range of motion of the right shoulder. Pain was elicited upon direct palpation of the joint and with both active and passive movement of the ipsilateral shoulder.

Initial laboratory analysis showed elevated inflammatory markers (Table 1), including an erythrocyte sedimentation rate (ESR) of 48 mm/hr (normal range < 15 mm/hr) and a C-reactive protein (CRP) of 58 mg/L (normal range <5 mg/L).

**Table 1:** Laboratory findings on admission (Day 0) and Day 10

Parameter	Admission (Day 0)	Day 10	Normal Range
White blood cells (WBC)	10.78 x10^3^/µL	7.87 x10^3^ /µL	4.8-10.8 x10^3^ /µL
Neutrophils	61 %	50 %	60-70 %
Erythrocyte sedimentation rate (ESR)	48 mm/hr	25 mm/hr	0-15 mm/hr
C-reactive protein (CRP)	58 mg/L	6 mg/L	0-5 mg/L

**Figure 2:**
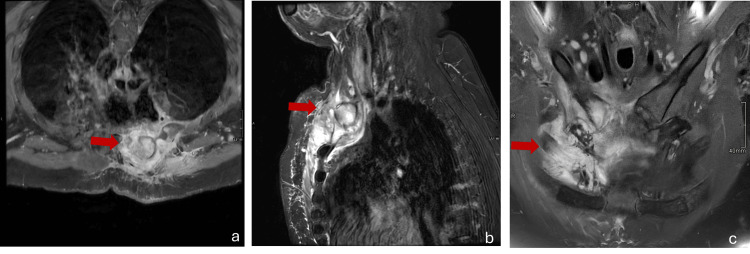
T2-weighted fat-saturated MRI of the right shoulder (a) Axial view, (b) sagittal view, and (c) coronal view Arrows indicate areas of osteomyelitis with associated cellulitis: 7x7 cm hyperintense area of inflammation in the axial plane (a) and 6.5 cm in the anteroposterior dimension on the sagittal plane (b) extending from the right pectoralis muscle through the costochondral junction of the first rib into the right internal mammary space.

MRI of the right shoulder demonstrated osteomyelitis with associated cellulitis (Figure 2). There was a 7x7cm area of inflammation in the axial plane of the right anterior chest wall extending from the right pectoralis muscle through the costochondral junction of the first rib, into the right internal mammary space, with the internal mammary vessels being lifted. It also revealed edema and erosions of the anterior arch of the first rib, distal right clavicle, right sternoclavicular junction, and manubrium.

The patient underwent CT-guided biopsy of the medial aspect of the right clavicular head, and soft tissue material was retrieved for pathological examination and microbiological culture. Representative bone tissue could not be obtained. He was subsequently started on intravenous linezolid and ceftazidime empirically for one week while awaiting culture results.

The blood culture showed no growth, whereas the tissue culture revealed multisensitive *Pseudomonas aeruginosa*, prompting the continuation of intravenous ceftazidime alone for an additional week (Table 2).

**Table 2:** Culture results and antibiotic susceptibility profile of the isolated organism

Specimen	Organism isolated	Antibiogram result
Blood	No growth after 8 days of incubation	―
Tissue	Pseudomonas aeruginosa	Sensitive to piperacillin-tazobactam, cephalosporins (ceftazidime, cefepime), carbapenems (meropenem, imipenem), monobactams, aminosides, quinolones (ciprofloxacin)

**Figure 3:**
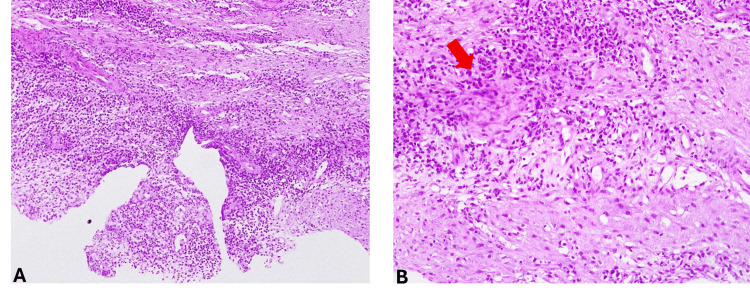
Histopathological examination showing nonspecific subacute inflammatory changes in the fibrous and adjacent striated muscle tissue obtained from right sternoclavicular junction biopsy (hematoxylin and eosin stain; original magnification x10 (A) and x40 (B)). A representative area of inflammatory infiltrate with seroleukocytic material is indicated by an arrow in the higher-magnification panel (B).

The histopathological analysis revealed nonspecific subacute inflammation, without any evidence of malignancy (Figure 3). Nevertheless, it was limited by the lack of bone tissue in the biopsy specimen. The examined sections showed fibrous tissue and striated muscle, with an inflammatory infiltrate and sero-leukocytic material. The fibrous tissue contains rare lymphoid aggregates and a polymorphous leukocytic infiltrate with mononuclear predominance. Immunohistochemistry was performed with appropriate external and internal positive controls, and it showed a polyclonal lymphocytic infiltrate, CD3-positive, CD20-positive, with a plasmacytic component, CD138-positive. 

Our patient improved clinically on medical therapy alone without requiring physical therapy, and CRP decreased from 58 to 6 mg/L after 10 days of intravenous antibiotics (Table 1). 

After completing a 2-week course of inpatient intravenous antibiotics, he was discharged home on ciprofloxacin 750 mg twice daily for 4 weeks.

## Discussion

A sternoclavicular joint infection represents less than 1% of all joint infections. The diagnosis is often delayed due to unusual presentation or prior treatment with steroids, resulting in the progression of septic arthritis to osteomyelitis [1]. It may occur via direct invasion from adjacent tissues or through hematogenous spread and subsequent joint seeding, although immunocompetent individuals are usually able to clear transient bacteremia [2,9,10]. Due to their low diagnostic yield, blood cultures cannot be solely relied on for the identification of the offending organism [11], and in our case, all sets of blood cultures did not yield any growth.

The sternoclavicular joint is characterized by a relatively non-distensible capsule; thus, the accumulation of a small amount of effusion secondary to an infectious process will lead to an increase in intra-articular pressure, promoting extension into adjacent structures and predisposing patients to serious complications such as mediastinitis and empyema [3,12].

The most common organism associated with sternoclavicular joint infection is *Staphylococcus aureus* [4], while *Pseudomonas aeruginosa* was mainly identified in intravenous drug users and patients requiring recurrent blood transfusions, notably those with thalassemia or sickle cell disease [5].

To our knowledge, this is the first case of sternoclavicular joint infection with *Pseudomonas aeruginosa* in a young immunocompetent patient concurrent with hidradenitis suppurativa and occurring in the absence of classic predisposing risk factors, including intravenous drug abuse, history of trauma, arthropathy, intra-articular injections, presence of a central venous catheter, or prior hospitalization [1]. In fact, patients with hidradenitis suppurativa tend to have a threefold higher risk of developing inflammatory arthritis, notably spondyloarthritis and rheumatoid arthritis, rather than septic arthritis [6]. Nevertheless, patients with uncontrolled or untreated hidradenitis suppurativa are susceptible to infectious complications secondary to immune system dysregulation, skin barrier disturbance, and bacterial colonization. Moreover, obesity, as observed in our patient case, may contribute to a proinflammatory state, thereby altering skin microbiota and leading to increased vulnerability to infections [7]. However, our patient currently has quiescent hidradenitis suppurativa without evidence of active inflammation during the six-month period preceding presentation.

Furthermore, the most frequently identified organisms in hidradenitis suppurativa lesions were *Staphylococcus* genus species, followed by *Corynebacterium*, *Streptococcus*, and anaerobic bacteria such as *Prevotella* and *Porphyromonas* species. In fact, *Corynebacterium* is among the main components of skin microbial flora, and it is preferentially present in intertriginous regions, including the axilla. On the other hand, *Pseudomonas aeruginosa *was comparatively far less common [8,13].

A previous study reported the first isolation of Pseudomonas Oryzihabitans, an opportunistic nosocomial agent, from HS lesions, although the patient had diabetes mellitus and a history of prior hospital admission [14].

This case highlights the possibility of sternoclavicular junction infection occurring spontaneously without risk factors. *Pseudomonas* infection should be considered even in immunocompetent patients. Furthermore, hidradenitis suppurativa may predispose to atypical infections through chronic inflammatory state, skin barrier disturbances, and breaks or subclinical bacteremia and subsequent joint seeding. Although the association between these clinical entities remains unclear, hidradenitis suppurativa should be considered as a potential risk factor for sternoclavicular joint infection, and it deserves further evaluation.

Therefore, it is crucial to maintain a high index of suspicion when evaluating young individuals for chest or shoulder pain. Early recognition and definitive diagnosis through biopsy are essential for appropriate management with antibiotic therapy in order to avoid complications requiring surgical therapy, ranging from simple incision with drainage and debridement to more radical interventions [3].

## Conclusions

Clinicians should consider a broad differential diagnosis when evaluating joint infections, even in young and immunocompetent individuals. Hidradenitis suppurativa may have predisposed to the development of a *Pseudomonas aeruginosa* sternoclavicular joint infection through recurrent bacteremia and subsequent joint seeding. This report can be added to the limited existing literature on *Pseudomonas* sternoclavicular osteomyelitis, and emphasizes the need to consider unusual pathogens in atypical anatomical sites, particularly in patients with chronic inflammatory skin diseases.
